# Studying subcellular detail in fixed astrocytes: dissociation of morphologically intact glial cells (DIMIGs)

**DOI:** 10.3389/fncel.2013.00054

**Published:** 2013-05-03

**Authors:** Julia Haseleu, Enrico Anlauf, Sandra Blaess, Elmar Endl, Amin Derouiche

**Affiliations:** ^1^Institute of Cellular Neurosciences, University of BonnBonn, Germany; ^2^Institute of Anatomy II, University of FrankfurtFrankfurt am Main, Germany; ^3^Dr. Senckenbergisches Chronomedizinisches Institut, University of FrankfurtFrankfurt am Main, Germany; ^4^Institute of Reconstructive Neurobiology, Life and Brain Center, University of BonnBonn, Germany; ^5^Institute of Molecular Medicine, University of BonnBonn, Germany

**Keywords:** actin, tubulin, ezrin, connexin 43, gap junction, synapse

## Abstract

Studying the distribution of astrocytic antigens is particularly hard when they are localized in their fine, peripheral astrocyte processes (PAPs), since these processes often have a diameter comparable to vesicles and small organelles. The most appropriate technique is immunoelectron microscopy, which is, however, a time-consuming procedure. Even in high resolution light microscopy, antigen localization is difficult to detect due to the small dimensions of these processes, and overlay from antigen in surrounding non-glial cells. Yet, PAPs frequently display antigens related to motility and glia-synaptic interaction. Here, we describe the dissociation of morphologically intact glial cells (DIMIGs), permitting unambiguous antigen localization using epifluorescence microscopy. Astrocytes are dissociated from juvenile (p13–15) mouse cortex by applying papain treatment and cytospin centrifugation to attach the cells to a slide. The cells and their complete processes including the PAPs is thus projected in 2D. The entire procedure takes 2.5–3 h. We show by morphometry that the diameter of DIMIGs, including the PAPs is similar to that of astrocytes *in situ*. In contrast to cell culture, results derived from this procedure allow for direct conclusions relating to (1) the presence of an antigen in cortical astrocytes, (2) subcellular antigen distribution, in particular when localized in the PAPs. The detailed resolution is shown in an exemplary study of the organization of the astrocytic cytoskeleton components actin, ezrin, tubulin, and GFAP. The distribution of connexin 43 in relation to a single astrocyte's process tree is also investigated.

## Introduction

The role of astrocytes for CNS synaptic transmission, including their own active signaling has become increasingly clear over the past years (Haydon and Carmignoto, 2006). Astrocytes are also key players in the coupling of metabolism and blood flow to neuronal activity (Carmignoto and Gómez-Gonzalo, 2010; Giaume et al., 2010). In addition to their soma and GFAP-positive main processes, astrocytes—in particular in gray matter—display a high amount of extremely fine processes. The astrocyte approaches the synaptic cleft, blood capillaries, the surface of neuronal somata and neuronal compartments (Reichenbach et al., 2004) mostly through these processes. These processes have been termed peripheral astrocyte processes (PAPs; Derouiche et al., 2002), and have been shown to preferentially display proteins and organelles involved in mediating glial interactions with neurons or vessels (e.g., Danbolt et al., 1998; Derouiche and Frotscher, 2001; Rouach et al., 2008). The PAPs, although emanating from the main processes, are best regarded as sponge-like structures rather than highly branched processes, based on electron microscopic 3D reconstructions (Ventura and Harris, 1999; Witcher et al., 2007). Frequently they fully engulf or embed neuronal structures, insulating them, and creating a micro-environment (Reichenbach et al., 2010).

Light microscopical analysis of glial cells, in particular of astroglial cells has lead to misinterpretations since the beginnings of glial research. Although stained PAPs can be detected by the light microscope they cannot be resolved from small neuronal structures such as spine necks, subplasmalemmal staining in dendrites, or the extracellular matrix. At the ultrastructural level, glial antigens localized preferentially within PAPs are reliably detected based on established morphological criteria (Peters et al., 1991). However, immunoelectron microscopy is a time-consuming and complex procedure.

The present paper suggests a technique facilitating the study of these processes, in particular antigen localization, by light microscopic immunocytochemistry in astrocytes. We describe a cell dissociation procedure, which preserves the structural integrity of cortical astrocytes, including their PAPs, to a very high degree. Acutely isolated astrocytes are mainly prepared for electrophysiological analysis, sometimes combined with molecular biology (Steinhäuser et al., 1994; Seifert and Steinhäuser, 1995; Schools and Kimelberg, 2001), which requires of course live cells. Phase contrast microscopy of live cells or *post-hoc* fixation and staining shows that those isolated astrocytes have collapsed or curled-back main processes, and reduced PAPs. Lopez et al. (1997), applying a proprietary enzyme for dissociation, focussed on structural preservation of fixed astrocytes and Müller cells, and obtained remarkable results as shown by the presence of many elongated main processes. These preparations are still not suitable to immunocytochemically investigate PAPs, which are often only 50–200 nm wide. The dissociation procedure described here allows for subcellular antigen localization even within the thinnest processes of glial cells. At the same time, many of the astrocytes dissociated and observed without surrounding cells still display their complex, elaborated morphology, which has prompted us to term both the procedure and astrocytes obtained Dissociation of/dissociated morphologically intact glial cells (DIMIGs).

## Materials and methods

Animal handling and sacrifice was carried out so as to minimize suffering, in accordance with animal welfare legislation. The optimized dissociation procedure consists of eight main steps (Table 1); for details and comments see next section.

**Table 1:** **Principal steps of the dissociation of morphologically intact glial cells (DIMIG)**.

**1. Cortex dissection (p13–p15)**
↓
**2. Cortex slicing**
↓
**3. Pre-incubation of cortical tissue**
↓
**4. Enzymatic dissociation with papain**
↓
**5. Mechanical dissociation with pipettes**
↓
**6. One-step density gradient centrifugation**
↓
**7. Cytospin centrifugation**
↓
**8. Fixation of cells on slide**

### Cytochemistry

After washing with PB, fixed dissociated cells were incubated sequentially with Triton X-100 (0.2% in PB, 2 min), normal serum (10% in PB, 30 min), primary antibodies (overnight), and secondary antibodies (1 h). Antibodies and concentrations are listed in Table 2. For nucleus localization, cells were incubated with bisbenzimidine (Sigma-Aldrich, Deisenhofen, Germany; 1:200,000, 30 min). Specimens were documented using a fluorescence microscope (Zeiss Cell Observer, Jena, Deutschland).

**Table 2:** **Summary of primary and secondary antibody combinations, and affinity labels and concentrations used**.

**Primary antibody (concentration)**	**Supplier/source**	**Secondary antibody (concentration)**
ck anti-GFAP (1:500)	Chemicon/Millipore, Billerica, USA	AMCA-coupled to dk anti-ck (1:100)
ms anti-GFAP-CY3 (1:2000)	Sigma-Aldrich, Deisenhofen, Germany	
shp anti-GFP (1:4000)	Serotec, Düsseldorf, Germany	DyLight488-coupled to dk anti-shp (1:100)
ms anti-ezrin (1:500)	Sigma-Aldrich, Deisenhofen, Germany	CY3-coupled to dk anti-ms (1:1000)
rb anti-ezrin (1:1000)	Upstate/Millipore, Billerica, USA	CY3-coupled to dk anti-rb (1:1000)
ms anti-α-tubulin (1:500)	Sigma-Aldrich, Deisenhofen, Germany	CY3-coupled to dk anti-ms (1:1000)
ms anti-ß-actin (1:500)	Sigma-Aldrich, Deisenhofen, Germany	CY3-coupled to dk anti-ms (1:1000)
rb anti-connexin 43	Sigma-Aldrich, Deisenhofen, Germany	CY3-coupled to goat anti-rb (1:1000)
Phalloidin-Oregon Green 488 (5 units/ml)	Molecular Probes/Invitrogen	

ck, chicken; dk, donkey; shp, sheep; ms, mouse; rb, rabbit.

### Morphometry and statistical analysis

Morphometric parameters of astrocytes were analyzed using the image analysis software ImageJ (Rasband, 1997, 1998, 1999, 2000, 2001, 2002, 2003, 2004, 2005, 2006, 2007, 2008, 2009, 2010, 2011). The following morphometric parameters were determined from individual astrocytes double-stained for GFAP (main processes) and ezrin (PAPs): astrocytic diameter (based on their longest main processes, GFAP-IR positive), length of the peripheral astrocytic processes (ezrin-IR positive), and the area fraction of PAPs in relation to the overall astrocytic area. To determine the diameter of astrocytes, the distance between the most distal tips of the two longest opposing main processes was measured. The mean length of the peripheral astrocytic processes was determined by measuring the distance between the point of origin of the PAPs from the main process, and their distal tips. To account for the variability in PAP length, five PAPs emanating from different main processes of each astrocyte were measured. To obtain the area fraction of PAPs in relation to the overall astrocyte surface, the area positive for GFAP was segmented, measured, and substracted from the area occupied by either one or both of the GFAP and ezrin labelings. Statistical analysis was performed using Excel (Microsoft, Redmond, WA, USA) and SPSS 13 (SPSS Inc., Chicago, IL, USA).

### High resolution microscopy

For immunofluorescence, sections were photodocumented using a 63 × 1.4 lens. To increase resolution, image stacks at 50–100 nm steps were recorded (Zeiss 200 M microscope), and deconvolved using calculated PSF and iterative deconvolution (Volocity Software, Perkin Elmer, Waltham, MA, USA). For colocalization studies, correction for residual chromatic aberration was applied in image stack construction (Anlauf and Derouiche, 2009).

## Results

### Dissociation procedure

The dissociation method developed here is based on previous cell isolation protocols (e.g., Steinhäuser et al., 1994; Kimelberg et al., 2000; Lu et al., 2006; Lovatt et al., 2007; Worthington Biochemical Corp., Lakewood, NJ, USA). The aim of the study was to optimize these protocols with respect to morphological preservation of the very thin glial processes.

Juvenile mice (age: postnatal day 13–15) were deeply anesthetized with isoflurane and sacrificed by decapitation; the brain was immediately removed. While floating in ice-cold sucrose-based aCSF (2.5 mM KCl, 25 mM NaHCO_3_, 87 mM NaCl, 1.25 mM NaH_2_PO_4_, 25 mM D-glucose, 0.5 mM CaCl_2_, 7 mM MgCl_2_, 75 mM sucrose; 330 mOsm; pH 7.4; pre-equilibrated with carbogen), the brain was carefully freed from meninges to reduce contaminating blood cells, and the cortices were extracted (Table 1, step 1). Cortical slices (300 μm) were cut in ice-cold sucrose-based aCSF (pH 7.4; 330 mOsm; pre-equilibrated with carbogen) using a vibratome (Leica VT1200S, Wetzlar, Deutschland; Table 1, step 2). Next, the slices were pre-incubated in aCSF for 30 min at 35°C, and subsequently in Ca^2+^/Mg^2+^-free EBSS (Sigma-Aldrich, Deisenhofen, Germany; equilibrated with carbogen) for 15 min at 22°C (Table 1, step 3). Ca^2+^/Mg^2+^-free EBSS is essential for preservation of cell morphology. It is used to loosen the extracellular matrix prior to enzymatic dissociation (Seifert and Steinhäuser, 1995), probably by reducing the action of the Ca^2+^-dependent cadherins present in brain tissue. To degrade the extracellular matrix completely, the slices were incubated in a carbogenated papain solution [20 units/ml papain, 1 mM L-cysteine, 0.5 mM ethylenediaminetetraacetate (EDTA) in Ca^2+^/Mg^2+^-containing EBSS; Worthington Biochemical Corp., Lakewood, NJ, USA] for 15 min at 37°C (Table 1, step 4). During this step, Ca^2+^/Mg^2+^-containing EBSS was used since the cysteine protease papain is Ca^2+^-dependent. After enzymatic treatment, the digested tissue was mechanically dissociated by gentle trituration (10 times), using a 10 ml pipette as used for cell culture media (Corning Inc., Corning, NY, USA). The resulting suspension was centrifuged for 6 min at 280 g, at 22°C (Table 1, step 5). Subsequently, the enzymatic reaction was stopped and extracellular DNA digested by resuspending the pellet in inhibitor solution (1 mg/ml ovomucoid, 1 mg/ml BSA, 0.0005% DNase I in Ca^2+^/Mg^2+^-containing EBSS; Worthington Biochemical Corp., Lakewood, NJ, USA). To remove debris and dead cells, a one-step density gradient centrifugation (70 g, 6 min, 22°C) was performed (Table 1, step 6). Five ml of concentrated inhibitor solution (10 mg/ml ovomucoid, 10 mg/ml BSA in Ca^2+^/Mg^2+^-containing EBSS) in a 15 ml plastic tube were covered by the cell suspension. After centrifugation, the supernatant was discarded, and the cells were resuspended (5–10 times trituration using a 1 ml disposable plastic micropipette tip) in 2 ml of Ca^2+^/Mg^2+^-containing EBSS (Worthington Biochemical Corp., Lakewood, NJ, USA) plus 1% BSA. To obtain a desired degree of cell separation on the slide, the resulting solution was further diluted to varying degrees (mostly 1:64). Finally, the cells were attached to positively charged slides (SuperFrost Plus, Thermo Fisher Scientific Inc., Waltham, MA, USA) using cytospin centrifugation (Shandon Cytospin 4 Cytocentrifuge, Thermo Fisher Scientific Inc., Waltham, MA, USA; 800 rpm, 10 min, 22°C) (Table 1, step 7) and immediately afterwards fixed with 4% paraformaldehyde in PB for 10 min at 22°C (Table 1, step 8). In order to adjust the desired cell-to-cell distance on the slide, the resuspension solution was appropriately diluted before cytospin centrifugation, with Ca^2+^/Mg^2+^-containing EBSS. After fixation, the slides were washed with PB and either immunostained or cryoprotected with increasing concentrations of sucrose solution (10, 20, and 30% sucrose in PB, 10 min each), snap-frozen and stored at −80°C.

**Figure 1:**
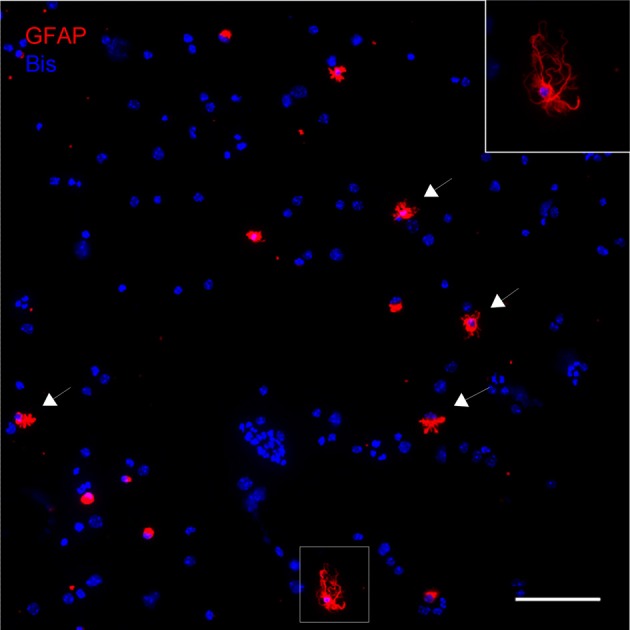
**Dissociated cells attached to a slide by cytospin centrifugation.** Astroctes are stained with anti-GFAP (red) and nuclei are stained with bisbenzimide (blue). Note that the majority of the cells are not GFAP positive, including any kind of neural cells or cells present in blood and vasculature. As an indication, nuclear size and morphology are highly variable. Astrocytes can be easily identified, many of them have well-preserved main processes (arrows). Inset: Higher magnification of astrocyte in boxed area. Scale bar: 100 μm.

The astrocytic fraction of the dissociated cells was not systematically measured. Figure 1 shows a representative image of dissociated GFAP-IR positive astrocytes that are attached to a slide by cytospin centrifugation. A large fraction of the dissociated astrocytes appears morphologically intact while others do not satisfy the criteria of good morphology.

### Morphometric analysis of dissociated astrocytes

**Figure 2:**
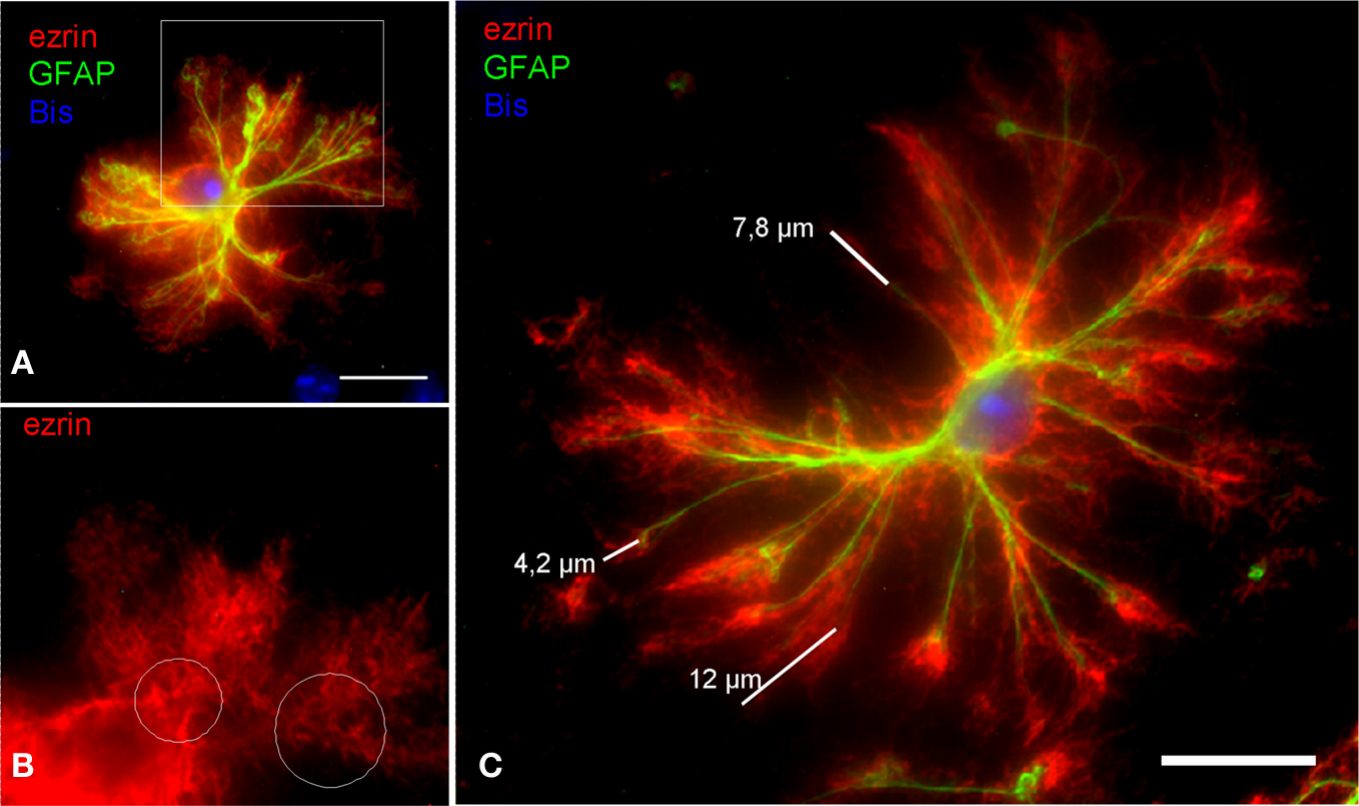
**In DIMIG astrocytes, the extensive astrocyte arborization is preserved in fine detail. (A)** Ezrin-IR positive PAPs (red) cover the entire astrocytic surface. **(B)** It is evident from a magnification of the boxed area in **(A)** that ezrin (red) mainly localizes to the PAPs. White circles indicate alleys poor in ezrin, which are occupied by the GFAP positive glial filament bundles of the main processes. This further illustrates that ezrin is a preferential PAP marker. **(C)** The ezrin positive PAPs (red) increase the stretch of the GFAP positive main processes (green) by as far as 12 μm, by a mean distance, however, of 6,64 μm. Scale bars: 15 μm.

The dissociated astrocytes frequently retain an extensive arborization of processes. Even the very thin PAPs, which are ezrin positive are preserved in fine detail (Figure 2). By applying morphometry we wanted to check whether the dissociation procedure yields morphologically fully intact astrocytes, or whether their processes tree is reduced in relation to astrocytes *in situ*. There are several *in situ* quantitations encompassing PAPs (e.g., Ventura and Harris, 1999; Witcher et al., 2007; for review see Reichenbach et al., 2010). We chose, however, to compare our findings to those of Chao et al. (2002), one of the very few reports selectively quantitating PAPs, i.e., in relation to glial main processes [for review see Wolff and Chao (2004)]. Chao et al. (2002) established their morphometric parameters in astrocytes *in situ* by analyzing specimens from rat cortex with electron microscopy.

The mean diameter of measured, dissociated astrocytes was 74.88 μm (SD = 27.01 μm; max = 129.96 μm; min = 35.33 μm; *n* = 20) and the reach of the main astrocytic processes was extended by PAPs on average by 4.65 μm (SD = 1.09 μm; max. = 6.64 μm; min. = 3.02 μm; *n* = 100). The corresponding diameter of rat astrocytes *in situ*, based on the main processes is 78 μm and the reach of the main processes via its PAPs is 4.85 μm (Chao et al., 2002). These values are comparable to the present measurements. We conclude that the dissociated astrocytes are preserved in nearly full detail.

### The astrocytic cytoskeleton in PAPs

**Figure 3:**
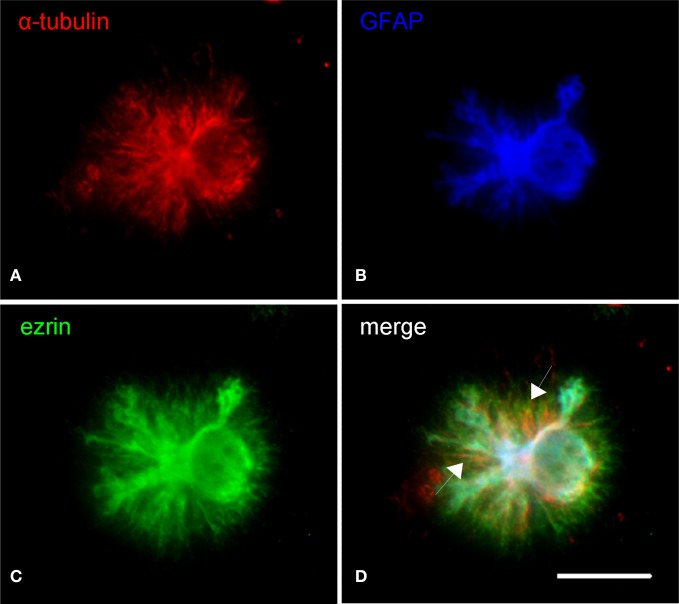
**Subcellular distribution of microtubules (α-tubulin, red) in relation to astrocytic main processes (GFAP, blue) and PAPs (ezrin, red). (A)** Microtubules mainly localize to the soma and main processes of astrocytes. A minor proportion of microtubules extends beyond the tips of the main processes **(B)**. Overlaying the channels illustrates that some microtubules even stretch to within the PAPs **(C**, arrows in **D)**. Scale bar: 15 μm.

**Figure 4:**
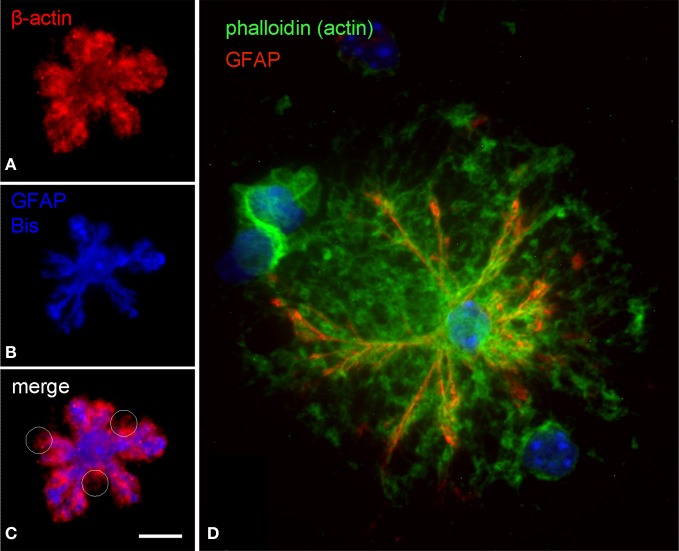
**Subcellular distribution of actin filaments in DIMIG astrocytes.** Actin was labeled by anti-actin (red in **A,C**) or by phalloidin coupled to Oregon-green **(D)**. Astrocytes display a dense meshwork of actin filaments, which distributes evenly all over the cell **(A,C,D)**. The actin meshwork clearly extends beyond the main processes (GFAP) and is strongly labeled also in the PAPs **(C,D)**. Scale bars: 10 μm **(A–C)**, 15 μm **(D)**.

The cytoskeleton composition of astrocytic processes, especially that of PAPs, is not well-studied and controversially discussed in the literature (Safavi-Abbasi et al., 2001; Hatton, 2002; Potokar et al., 2007; Peng et al., 2008; Kreft et al., 2009; Ou et al., 2009). To our best knowledge, this is the first time that cytoskeletal components are unambiguously localized in the different compartments of *in situ* like astrocytes. As shown in triple stainings the cytoskeleton of main astrocytic processes is composed of microtubules (anti-a-tubulin), intermediate (glial) filaments (anti-GFAP), and microfilaments (phalloidin, anti-actin; Figures 3, 4). PAPs almost exclusively and abundantly contain microfilaments (Figures 3, 4) with some microtubules extending into only their proximal parts (Figure 3).

### Gap junctions in PAPs

**Figure 5:**
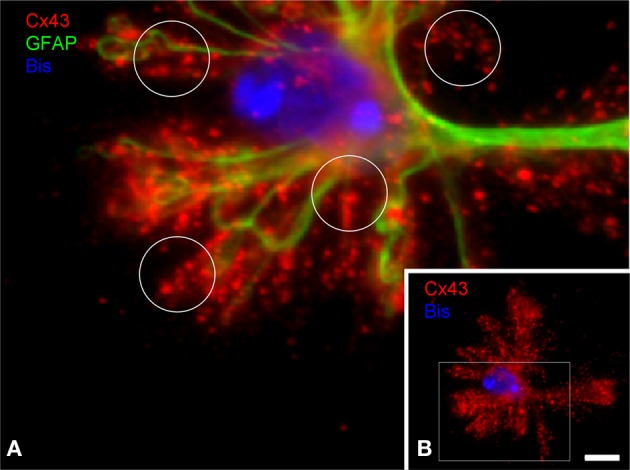
**Subcellular distribution of connexin 43 (C×43) in DIMIG astrocytes. (A)** Higher magnification of boxed area in **(B)**. C×43 is present in the perinuclear region (bisbenzimidine, blue for nucleus), as well as in the GFAP positive main processes and the PAPs (circles in **A**). C×43 distributes evenly to the three compartments soma, main processes, and PAPs **(B)**. Scale bar: 10 μm.

Cx43, the major astrocytic connexin, can form gap junctions and constitutes the molecular basis for interastrocytic coupling and for glial networks (Giaume et al., 2010). However, it is not clear how astroglial gap junctions are distributed within the individual astrocyte territory. Based on stainings in brain sections it cannot be concluded whether gap junctions are established only at the boundaries of the territory where the astrocyte contacts its neighbors. We thus studied the distribution of connexins in the individual astrocyte by immunostaining of DIMIGs. Cx43 was localized preferentially to the PAPs but only to a minor degree at the main processes (Figure 5), in the punctate manner known from tissue sections. Importantly, the Cx43 positive puncta were evenly distributed all over the astrocyte, including its more central, perinuclear portions.

## Discussion

### Dissociation of morphologically intact glial cells

We describe here a protocol optimized for the acute dissociation of morphologically well-preserved cortical astrocytes from juvenile (p13–15) mouse brain. Several parameters like (1) incubation time and (2) temperature, (3) ionic composition and (4) osmolarity of media, (5) the way of mechanical dissociation and (6) centrifugation had to be optimized. In particular, tight control of parameters (1)–(6) appears essential for obtaining a relatively high yield of sufficiently well-preserved astrocytes suitable for subcellular antigen localization. The proportion of well-preserved cells varies from slide to slide, and is roughly estimated 10–20% (cf. Figure 1) depending on which degree of morphology is considered acceptable for immunocytochemistry. Amongst the criteria for good, acceptable morphology are the presence of a few or several long main processes, which are also non-collapsed. In addition, PAPs are a clear indication of intact morphology, in particular PAPs extending from the distal parts of main processes, where they would be assumed to be torn first. When appreciating the shape of well-preserved astrocytes, one has to bear in mind that cells from all cortical layers are expected including various sizes of gray matter astrocytes, and fibrous white matter astrocytes.

A routine method in medical cytology, the cytospin centrifuge (Shandon Cytospin 4 Cytocentrifuge, Thermo Fisher Scientific Inc., Waltham, MA, USA) is definitely a key feature of the DIMIG—preparation. It greatly contributes to the outstretching of the astrocytes with their main and peripheral processes intact. This is a prerequisite for the visualization of the PAPs (Figure 2), which would otherwise be collapsed together with the main processes. Importantly, the DIMIG—procedure described here yields many other cell types present in live brain tissue, including those of blood and vessel wall, which are spun down together with astrocytes (see Figure 1). Although this remains to be demonstrated, we expect that structurally well-preserved cells of all other glial types can be found as well, since with this method antigens have been localized even in the fine and long processes of NG2 cells (Haberlandt et al., 2011). There is, thus, no isolation, purification, or enrichment of astrocytes, and consequently astrocyte-directed biochemistry or molecular biology cannot be performed.

DIMIG astrocytes appear as a valuable tool, since they combine several major advantages. The astrocytes are preserved in nearly full detail, as suggested by qualitative assessment of their main and peripheral processes (e.g., Figure 2), and morphometric validation (see below). Although DIMIG astrocytes frequently display long PAPs extending even from the distal end of the main processes (see Figure 2), it would not be realistic to assume that these astrocytes are 100% spared by the DIMIG procedure. They should not be taken as a 1:1 morphological representation of astrocytes *in situ*. The morphology of DIMIG astrocytes can be astonishing, but they should also not be used for morphometric characterization of *in situ* astrocytes, in particular since the dissociated cells are projected onto one plane. No three dimensional information can be obtained from DIMIG prepared astrocytes. Using DIMIGs, glial process architecture may only be analyzed with a limited set of parameters, such as process length, number, and distance of branching points. More detailed description encompassing e.g., process angles, relative density of PAPs, and volumenometric parameters clearly will have to be based on astrocytes *in situ*. Similarly, the glial relation to specific neighboring structures, such as synapses, neuronal compartments, and blood vessels cannot be analyzed in DIMIGs, since disconnecting them is the primary aim of dissociation.

An advantage of dissociation, however, this procedure leads to very high signal-to-noise ratio after labeling. Prior to cytospin centrifugation, the cell suspension is diluted to yield clearly separated cells, without surrounding cellular structures, or other background, which further aids unequivocal antigen detection, and subcellular colocalization studies in subresolution structures such as PAPs. Subcellular (co)localization has been carried out in primary culture, where vesicular antigens can be localized at the single orgenelle level (Coco et al., 2003; Anlauf and Derouiche, 2005) or in membrane sheets (Lang et al., 2002). These labeling and microscopy techniques, unsuitable for the tissue section, can now be applied to DIMIGs, an astrocyte preparation much closer reflecting the *in vivo* situation than primary astrocytes.

As another advantage over studying tissue sections by light or electron microscopy, DIMIGs also permit regarding the individual astroglial cell as a unity of observation. To study differential antigen localization in astrocytic compartments—soma, main processes, PAPs—the method of choice has been electron microscopic examination of astrocytes *in situ*, which is very laborious and time-consuming. The method described here greatly facilitates investigations on antigen distribution in relation to the individual astrocyte as a whole cell. Even using electron microscopy, this is possible only by statistical conclusion and within demanding projects (Wolff and Chao, 2004). For example, in DIMIGs, main and peripheral processes can easily be segmented by using multiple labeling and image processing, thus enabling object or compartment-oriented morphometry and densitometry (Figure 2).

Based on the maturation of the extracellular matrix and cell shapes, the structural preservation after dissociation highly depends on the animal age and to a lesser extent on the CNS region. Enzymatic digestion times and further incubation parameters have to be optimized for each postnatal age and region (cf. Kaneda et al., 1988; Lopez et al., 1997), implying that the protocol presented here specifically relates to dissociation of p13–15 mouse cortex. Within this age range, no obvious, systematic variability was observed. Although the animal age at which the cells are prepared (p13–15) is not fully adult, it can be classified as juvenile. Like for many electro-physiological investigations on astrocytes, this age has been chosen as a compromise between CNS maturation and cell viability; the resulting morphology can thus be compared to extant physiological data (Verkhratsky and Steinhäuser, 2000). The protocol given has been systematically developed by optimizing the parameters and checking for consistency. We found that deviating from the protocol substantially decreases the quality of the dissociated cells. In particular, severe cell swelling or cell lysis were found to result from variations in osmolarity, which needs to be tightly checked. For the trituration step, the amount of pipetting and the pipette have to be standardized. This step is an important variable in the actual process of cell separation, an optimum between minimizing both, the amount of persisting cell clusters and cell damage. Temperature control is a less rigid parameter, which further increases the quality of both cell separation and cell integrity.

By applying the DIMIG procedure described here, morphologically intact astrocytes can be obtained within less than 3 h, from anesthetizing the animal until fixation of the cytospinned cells. It is assumed that major changes in protein expression levels, antigen localization, or organelle distribution are unlikely to occur within this time period. For example, it is hard to conceive a short-term redistribution of gap junctions at the scale of a cell's territory. Other, more rapid processes such as membrane internalization, vesicle fusion, or rapid process motility (Cornell-Bell et al., 1990; Lavialle et al., 2011) might induce differences with regard to *in vivo* astrocytes. However, the DIMIG astrocytes are considered to be *in situ* like and to represent the morphology as well as the immunocytochemical phenotype of astrocytes *in situ*. As seen even at low magnification the dissociated astrocytes display many different shapes. Some GFAP positive astrocytes appear round or clumped (see Figure 1), with even the main processes collapsed or torn-off, obvious signs of compromised structural preservation. Also those astrocytes classified structurally well-preserved are morphologically heterogeneous. Considering that cortex was dissociated with all regions and layers including white matter, this may reflect the spectrum of astrocytic morphologies observed in the cortex *in situ*. Thus, GFAP positive DIMIGs were reminiscent of the classical, star-shaped astrocyte, but also of astrocytes in white matter, the multiform or molecular layers, or those apposed to blood vessels, which all differ in size, process pattern, and/or territorial shape. As a logical consequence of the dissociation method, the putative laminar origin of the individual DIMIG astrocyte cannot be verified.

Previous dissociation and isolation procedures for astrocytes mainly focus on purity and viability rather than morphology of cells to enable biochemical, single-cell RT-PCR, and electrophysiological experiments (Steinhäuser et al., 1994; Seifert and Steinhäuser, 1995; Schools and Kimelberg, 2001). These previously described astrocyte dissociations are based on the method described by Kimelberg et al. (2000). It is not optimized for morphological integrity, since shearing-off of processes at 15 days postnatal (in rat) was consistently noted (Kimelberg et al., 2000). In particular, in combination with cytoplasmic GFP fluorescence in GFP-GFAP astrocytes, that method could still be used to verify astrocyte-specific localization of putatively neuronal proteins e.g., synaptotagmin IV (Zhang et al., 2004a) or several other vesicular proteins implicated in astrocytic glutamate release (Zhang et al., 2004b). Working on retinal (Müller) glial cells, Reichenbach et al. (1990) succeeded in preparing dissociated cells with preserved resting membrane potential, by reducing papain digestion to 20 min. Inspite of apparently torn-off lateral processes (corresponding to PAPs), these Müller cells had good ultrastructure. Because of differing solutions and concentrations, the papain digestion parameters of Reichenbach et al. (1990) and ours cannot be directly compared. In agreement with the findings of Reichenbach et al. (1990), we were able to minimize papain digestion time to 15 min, at the same time permitting a sufficient degree of dissociation. To our knowledge the only dissociation method aiming at morphological integrity of astrocytes (and Müller cells) is that described by Lopez et al. (1997). Unfortunately, their enzyme used for dissociation has not been available, and the main focus of that study was the preservation of glial main processes. Primary astrocyte culture, a standard model in glial research, serves as a good tool to obtain first hints concerning astrocytes *in situ*, but may display marked differences in relation to them. For example, cultured astrocytes abound with organelles positive for the vesicular glutamate transporters 1 and 2 (Anlauf and Derouiche, 2005), whereas they are hard to detect and have long been overlooked in astrocytes *in situ* (Bergersen and Gundersen, 2009).

### Cytoskeleton and connexin 43 in PAPs

The GFAP positive processes of DIMIGs also contain microtubules and actin microfilaments, which was to be expected. The PAPs are particularly rich in microfilaments, which is also seen in the filopodia of cultured astrocytes (Lavialle et al., 2011). Only occasionally, PAPs have microtubules, which when present reach only into their proximal parts but not into their tips. Such compartment specific findings, again, would be hard to assess using electron microscopy, which has much higher resolution but a more limited scope of observation relating to the individual cell. Reflecting the multiple functions of PAPs in glia-neuronal and glia-vascular communication, many of the corresponding proteins are preferentially targeted to the PAP, although the underlying mechanisms are unknown. With the presently observed high content in actin and the occasional presence of microtubules in PAPs, it might be speculated that transport organelles or protein complexes move along the microtubule to at least the base of the PAP, where the cargo is transferred to actin-based transport mechanisms. Ezrin, an ERM protein first associated with tethering the cell membrane to the actin cytoskeleton (Tsukita et al., 1997; Gautreau et al., 2002), and known to be present in PAPs (Derouiche and Frotscher, 2001), might also contribute in this process.

Orthogonal arrays of particles (OAPs) in glial cells, which are known to correspond to connexins, disappear after long papain treatment but remain in a localization comparable to *in situ* specimens after mild papain treatment (Reichenbach et al., 1990). The minimized papain digestion applied in the DIMIG procedure, and the finding of the well-known punctate pattern in Cx43 immunostained DIMIGs strongly indicate that the subcellular connexin localization is maintained after dissociation. Cx43 immunostaining in DIMIGs displays the well-known punctate pattern. It is interesting to note, however, that the puncta do not directly delineate the main processes of the individual astrocyte but rather form “clouds” of puncta, representing gap junctions established by the PAPs. It has been calculated from ultrastructural material that an average (rat cortical) astrocyte carries 30,000 gap junctions, and that 4000 of them are engaged in intra-astrocytic coupling, i.e., autocoupling (Wolff and Chao, 2004), a phenomenon also seen in primary astrocyte culture (Wolff et al., 1998). The present DIMIG preparation would be the first way to visualize the great extent of autocellular coupling, since CX43 positive puncta are dense also in the more central and perinuclear part of the territory.

In summary, the DIMIG procedure described here combines the microscopic advantages of primary astrocytes (high resolution, very high signal-to-noise ratio) with the properties of *in situ* like dissociated astrocytes. It can be used to overcome several disadvantages of the tissue section, primary astrocyte culture, and previously described dissociation procedures. Various antigens can be unambiguously localized, even within the thinnest, subresolution processes of astrocytes by applying conventional light microscopy. Maybe this preparation can be further exploited and developed at the stage prior to fixation to study, in viable astrocytes, the physiology of processes.

### Conflict of interest statement

The authors declare that the research was conducted in the absence of any commercial or financial relationships that could be construed as a potential conflict of interest.
